# Ultrasound assessment of humeral shaft nonunion risk: a feasibility and proof of concept study

**DOI:** 10.1007/s00590-023-03725-5

**Published:** 2023-09-29

**Authors:** William M. Oliver, Jamie A. Nicholson, Katrina R. Bell, Thomas H. Carter, Timothy O. White, Nicholas D. Clement, Andrew D. Duckworth, A. Hamish R. W. Simpson

**Affiliations:** 1 Edinburgh Orthopaedics, Royal Infirmary of Edinburgh, 51 Little France Crescent, Edinburgh, EH16 4SA UK; 2 Centre for Population Health Sciences, Usher Institute, University of Edinburgh, 49 Little France Crescent, Edinburgh, EH16 4SB UK; 3 Department of Orthopaedics and Trauma, University of Edinburgh, 49 Little France Crescent, Edinburgh, EH16 4SB UK

**Keywords:** Humeral shaft, Fracture, Ultrasound, Callus, Nonunion prediction

## Abstract

**Purpose:**

To determine the feasibility and reliability of ultrasound in the assessment of humeral shaft fracture healing and estimate the accuracy of 6wk ultrasound in predicting nonunion.

**Methods:**

Twelve adults with a non-operatively managed humeral shaft fracture were prospectively recruited and underwent ultrasound scanning at 6wks and 12wks post-injury. Seven blinded observers evaluated sonographic callus appearance to determine intra- and inter-observer reliability. Nonunion prediction accuracy was estimated by comparing images for patients that united (*n* = 10/12) with those that developed a nonunion (*n* = 2/12).

**Results:**

The mean scan duration was 8 min (5–12) and all patients tolerated the procedure. At 6wks and 12wks, sonographic callus (SC) was present in 11 patients (10 united, one nonunion) and sonographic bridging callus (SBC) in seven (all united). Ultrasound had substantial intra- (weighted *kappa*: 6wk 0.75; 12wk 0.75) and inter-observer reliability (intraclass correlation coefficient: 6wk 0.60; 12wk 0.76). At 6wks, the absence of SC demonstrated sensitivity 50%, specificity 100%, positive predictive value (PPV) 100% and negative predictive value (NPV) 91% in nonunion prediction (overall accuracy 92%). The absence of SBC demonstrated sensitivity 100%, specificity 70%, PPV 40% and NPV 100% in nonunion prediction (overall accuracy 75%). Of three patients at risk of nonunion (Radiographic Union Score for HUmeral fractures < 8), one had SBC on 6wk ultrasound (that subsequently united) and the others had non-bridging/absent SC (both developed nonunion).

**Conclusions:**

Ultrasound assessment of humeral shaft fracture healing was feasible, reliable and may predict nonunion. Ultrasound could be useful in defining nonunion risk among patients with reduced radiographic callus formation.

## Introduction

Humeral shaft fractures comprise 1% of all adult fractures [1] with an annual incidence of 12 per 100,000 [2]. Non-operative management is often favoured for patients with isolated, closed humeral shaft fractures, although nonunion may complicate one in six injuries managed in this way [3–5]. Early detection of patients at risk of humeral shaft nonunion using clinical [6] and/or radiological indicators [7] is assuming increasing importance, given the potential impact of nonunion upon longer-term patient-reported outcomes [8, 9].

Ultrasound assessment of the callus formation is emerging as a useful tool to assess fracture healing, with distinct advantages over radiographs including being non-invasive, avoiding ionising radiation and detecting fracture callus at an earlier stage [10]. Ultrasound has previously demonstrated potential in monitoring healing of humeral fractures after internal fixation [11], femoral and tibial fractures after external fixation [12] and tibial fractures after intramedullary nailing [13–15] or external fixation [16]. The role in monitoring healing after nonoperatively managed fractures has also been suggested for the clavicle [17, 18], femur and tibia [19]. However, the authors are aware of only one study relating to nonoperatively managed humeral shaft fractures, involving six patients [19]. Existing studies of ultrasound in the context of humeral shaft fracture healing are limited by incomplete reporting of the scanning technique and absence of data on the reliability and reproducibility of ultrasound image interpretation [11, 19]. Prospective validation of the feasibility of humeral shaft ultrasound, and the reliability in assessing fracture healing, is essential as a foundation for potential future use in clinical practice.

The primary aim of this pilot study was to determine the feasibility and reliability of ultrasound in the assessment of humeral shaft fracture healing. The secondary aim was to estimate the accuracy of six-week ultrasound assessment in predicting subsequent humeral shaft nonunion.

## Materials and methods

### Study cohort

The use of musculoskeletal ultrasound for the assessment of fracture healing received prospective Research Ethics Committee approval (reference number 06/S1103/51). Inclusion criteria for this study were: adult patients (aged ≥ 16 years); isolated, closed fracture of the humeral diaphysis; patients undergoing initial non-operative management (≥ 12 weeks post-injury); and patients able to provide informed, written consent to participate. Exclusion criteria were: pathological or periprosthetic fractures; re-fractures; open injuries; associated injuries/polytrauma; patients who underwent operative fixation within 12 weeks of injury and non-residents. From September 2018 to July 2019, 117 patients were prospectively identified from Emergency Department attendances and Trauma Triage/Fracture Clinic referrals, and 45 met the inclusion and exclusion criteria.

**Fig. 1:**
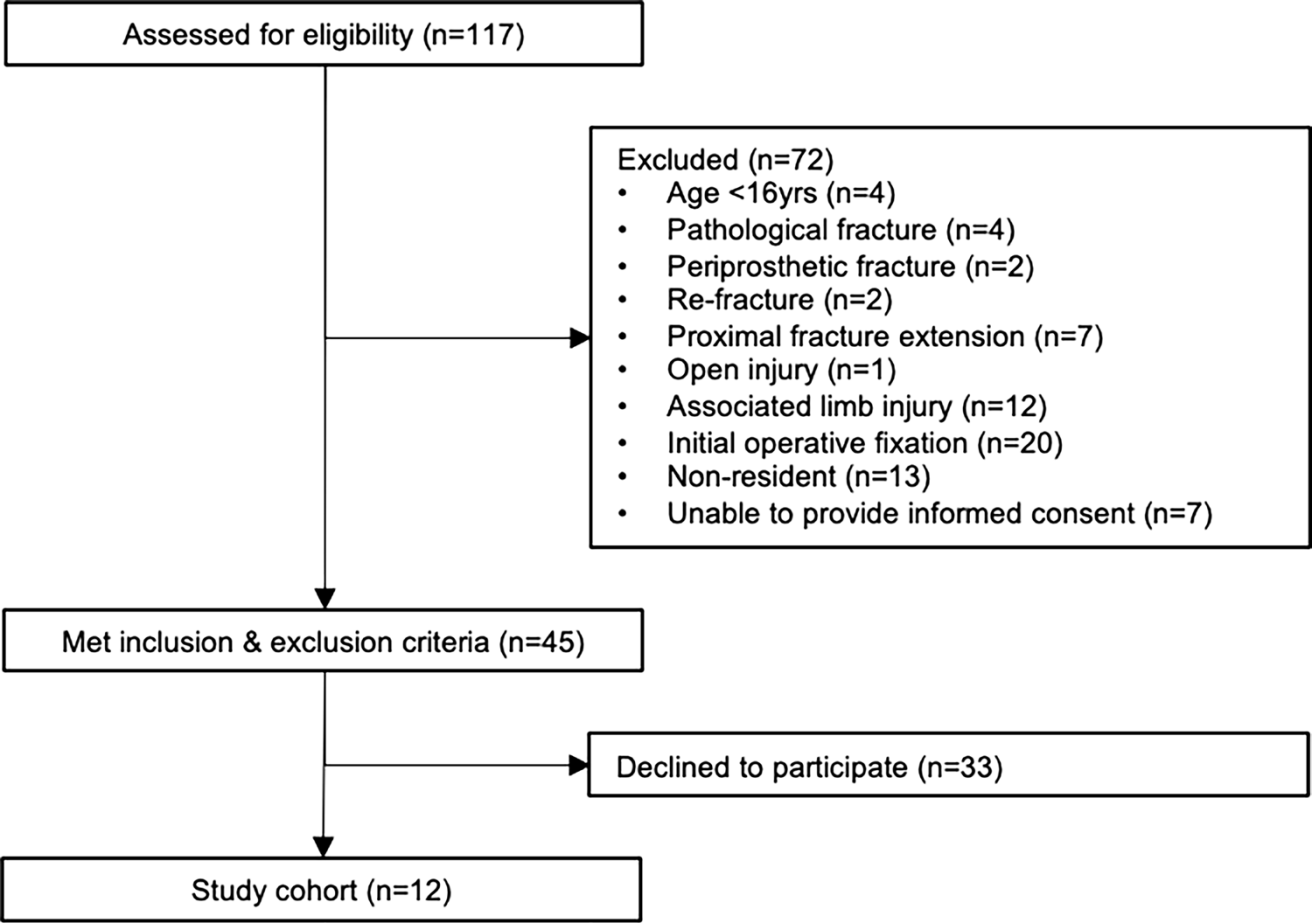
Identification of the study cohort

All patients were approached at their first outpatient clinic appointment and invited to participate, and those who provided informed consent (*n* = 12, 27%) were recruited (Fig. 1). Patients who declined (*n* = 33, 73%) generally did so based on a belief that scans would be painful and/or a desire to avoid prolonging their follow-up appointments.

### Patient and injury characteristics

Patient demographics, medical/social background and injury details were determined prospectively. A fall from height was defined as any height greater than six feet. Fractures were categorised according to location (proximal, middle, distal) and the AO-Orthopaedic Trauma Association (AO-OTA) classification [20]. There were no differences between patients that were excluded from or declined participation (*n* = 105) and the study cohort (*n* = 12) in terms of age (*p* = 0.403, Mann–Whitney U test), sex (*p* = 0.937, Chi-squared test [CS]) socioeconomic deprivation [21] (*p* = 0.818, CS), side of injury (*p* = 0.547, CS), fracture location (*p* = 0.505, CS) or AO-OTA fracture type [20] (*p* = 1, Fisher’s exact test).

### Management and outcome

All patients were placed into a plaster of Paris ‘U-slab’ in the Emergency Department, which was replaced by a functional brace following outpatient review within two weeks of injury. Clinical and radiographic follow-up was performed at six- and 12-week post-injury. Radiographic callus formation was prospectively assessed on anteroposterior and lateral radiographs at six weeks post-injury, according to the Radiographic Union Score for HUmeral fractures (RUSHU) [7]. Union was defined as reduced/absent pain at the fracture site, accompanied by bridging callus across all fracture cortices prior to clinic discharge [22, 23]. Nonunion was defined as a failure of the fracture to unite after 12 weeks of non-operative management, with the requirement for subsequent nonunion surgery [24, 25].

### Ultrasound assessment

Ultrasound scans were performed by a single orthopaedic surgeon with basic training and experience in musculoskeletal ultrasound. Scans were performed in the outpatient department alongside usual clinical and radiographic follow-up. Following clinical and radiographic assessment, patients were placed into a semi-recumbent position and their humeral brace was removed. The patient’s upper arm was placed into neutral rotation, with the forearm resting on a pillow. Scans were performed with the TA Sonix L14-5 MHz/38 mm ultrasound probe (BK Medical North America, Burlington, MA), using a standard setting for superficial musculoskeletal ultrasound evaluation (frequency 3 to 7 MHz, penetration depth 6 cm) [17]. Ultrasound images were obtained through two acoustic windows: The anterolateral window, in which the ultrasound beam was directed through the anterolateral fibres of the deltoid insertion (proximally), the fibres of brachialis at the lateral edge of the biceps brachii (mid-shaft) and within the brachialis/brachioradialis interval (distally); and the anteromedial window, in which the beam was directed within the deltopectoral groove (proximally), moving medially to the medial edge of the biceps brachii (mid-shaft and distally), passing through the medial fibres of brachialis at its most distal extent. Using these two acoustic windows, a 270-degree assessment of the humeral shaft was possible with only the posterior surface not visualised.

The fracture site was imaged in both longitudinal (long-axis) and axial (short-axis) planes. Multiple frames were captured over the site of interest, with the ultrasound probe carefully tilted and rotated into a variety of trajectories to ensure complete visualisation of callus and avoid anisotropy. The duration of the ultrasound scan (from start of long-axis image acquisition to the end of short-axis image acquisition) was recorded, and any technical challenges encountered during the procedure were documented. The tolerability of ultrasound scanning from the patient’s perspective was also recorded, in terms of reported discomfort and the need to abandon the procedure. The criteria for the two-dimensional detection and interpretation of sonographic callus have been described previously [17]. Scan images for observer assessment were rendered using Stradwin version 6.0 (Cambridge University Engineering Department, Cambridge, UK).

### Reliability and nonunion prediction

Ultrasound scan images were reviewed by seven orthopaedic surgeons (four registrars/residents, three consultants/attendings), blinded to the scan timing (six/12 weeks) and patient outcome (union/nonunion). Of these seven observers, two had prior experience of musculoskeletal ultrasound, while five did not. All observers were asked to evaluate the presence of sonographic callus as either ‘absent’, ‘non-bridging’ or ‘bridging’. To determine intra-observer reliability, a repeat set of blinded observations were obtained at least four weeks following the initial observations. The accuracy of ultrasound assessment in nonunion prediction was estimated by comparing ratings of scans for patients that united with those that developed a nonunion. Patients with reduced radiographic callus formation at six weeks post-injury, defined as a Radiographic Union Score for HUmeral fractures (RUSHU) < 8, were considered to be at increased risk of nonunion [7].

### Statistical analysis

Intra-rater reliability was determined using the weighted *kappa* statistic and 95% confidence interval (CI). For inter-observer reliability, a ‘single measures’ intraclass correlation coefficient (ICC) and 95% CI was calculated, using a two-way mixed model with assessment of consistency between observers. Reliability was interpreted as follows: 0–0.2 indicates poor agreement; 0.21–0.4 indicates fair agreement; 0.41–0.6 indicates moderate agreement; 0.61–0.8 indicates substantial agreement and more than 0.8 indicates near-perfect agreement [26]. Significance was set at *p* < 0.05.

## Results

### Cohort summary

Twelve patients were recruited into the study: The mean age was 54 years (range 20–81) and seven (58%) were female (Table 1). Of four patients with a concomitant radial nerve palsy at presentation, all recovered spontaneously at a median of nine weeks (range 2–26). Following non-operative management, ten patients (83%) united and two (17%) developed a nonunion. Both patients with a nonunion underwent successful nonunion surgery (plate and screw fixation) at 19 and 37 weeks post-injury, respectively.

**Table 1:** Baseline patient and injury characteristics for the study cohort (*n* = 12)

*Sex (n, %)*	
Male	5, 42%
Female	7, 58%
*Age at injury (years)*	
Mean ± SD	54.3 ± 20.2
Range	20.1–81.6
Median (IQR)	58.8 (43.8–68.9)
*Comorbidities (n, %)*	
None	5, 42%
≥ 1	7, 58%
*Smoking (n, %)*	
Never smoker	3, 25%
Ex-smoker	4, 33%
Current smoker	5, 42%
*Alcohol intake (n, %)*	
Abstinent	2, 17%
Moderate	7, 58%
Excess	3, 25%
*BMI (kg/m*^2)^	
Mean ± SD	27.0 ± 3.9
Range	22.3–34.9
Median (IQR)	25.7 (24.0–29.9)
*SIMD quintile*	
1 (most deprived)	2, 17%
2	3, 25%
3	1, 8%
4	2, 17%
5 (least deprived)	4, 33%
*Injury mechanism (n, %)*	
Fall from standing	6, 50%
Fall from height	2, 17%
Direct blow	1, 8%
Road traffic accident	1, 8%
Sport	1, 8%
Other	1, 8%
*Side of injury (n, %)*	
Right	6, 50%
Left	6, 50%
*Side of injury (n, %)*	
Dominant	4, 33%
Non-dominant	8, 67%
*Fracture location (n, %)*	
Proximal	3, 25%
Middle	5, 42%
Distal	4, 33%
*AO-OTA group (n, %)*	
A	9, 75%
B	3, 25%
*Radial nerve palsy (n, %)*	
None	8, 67%
Incomplete/partial	3, 25%
Complete	1, 8%

*AO-OTA Arbeitsgemeinschaft für Osteosynthesefragen*-Orthopaedic Trauma Association; *BMI* body mass index; *IQR* interquartile range; *SIMD* Scottish Index of Multiple Deprivation

^*^Significant at the *p* < 0.05 level

### Feasibility and patient-acceptability

The mean scan duration was eight minutes (range 5–12). Spiral fracture configurations (AO-OTA group A1/B2, *n* = 8), involving a greater length of the humeral shaft compared with other fracture types (AO-OTA groups A2/A3, *n* = 4), required an increased scan duration (mean duration for AO-OTA A1/B2 = 9 min, mean duration for AO-OTA A2/A3 = 5.5 min; *p* = 0.001, *t*-test). Three patients in the study cohort were classified as obese (BMI ≥ 30 kg/m^2^). Although an excessive soft tissue envelope was encountered in these patients, it was still possible to obtain good quality images.

All patients tolerated the ultrasound scanning procedure. Seven patients (58%) experienced mild discomfort during the six-week scan, and four (33%) during the 12-week scan. However, it was not necessary to abandon any scans due to patient discomfort, and there were no sex or age differences in scan tolerability. Although gentle external rotation of the humerus was sometimes required to access the anteromedial window adequately, all patients were able to tolerate this without significant discomfort.

### Reliability

**Fig. 2:**
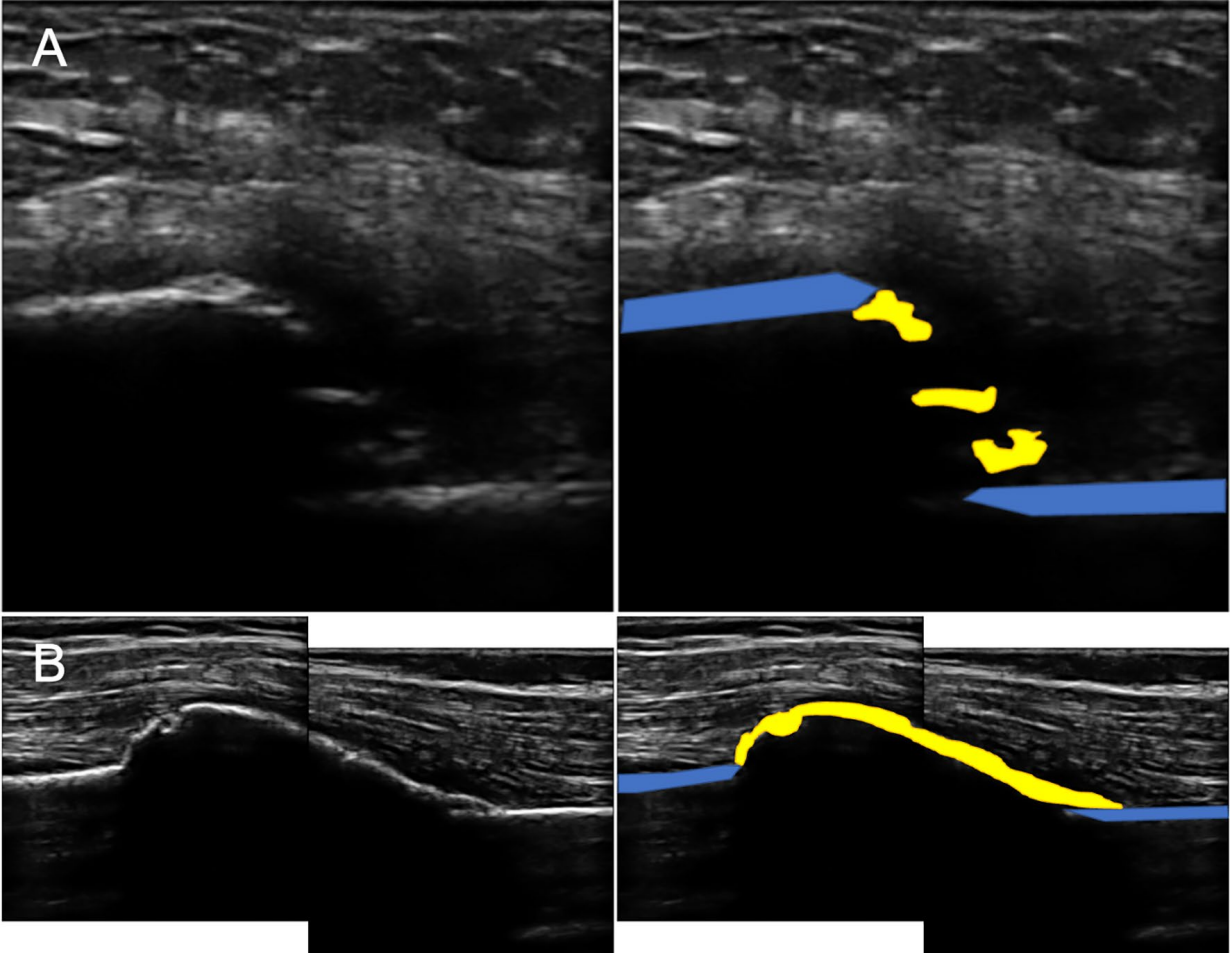
Ultrasound scan images obtained at six weeks following a humeral shaft fracture, showing: **A** Non-bridging callus; **B** Bridging callus; blue = bone cortex, yellow = sonographic callus

At both six and 12 weeks, sonographic callus (SC, either non-bridging or bridging) was present in 11 patients (10 united, one developed a nonunion) and sonographic bridging callus (SBC) was present in seven patients (all united; Table 2 and Fig. 2). Ultrasound image assessment demonstrated substantial intra-observer reliability, with *kappa* 0.75 (95% CI 0.47–1.03) at six weeks and 0.75 (95% CI 0.46–1.04) at 12 weeks. Inter-observer reliability of ultrasound image assessment was also substantial, with ICC improving from 0.60 (95% CI 0.38–0.83) at six weeks to 0.76 (95% CI 0.58–0.91) at 12 weeks.

**Table 2:** Radiographic (RUSHU) and ultrasound assessment of humeral shaft fracture healing at six weeks post-injury

Patient	Age/sex	Fracture location	AO-OTA group	RUSHU	Sonographic callus	Sonographic callus appearance	Outcome
1	30/M	Distal	A1	8	Present	Bridging	Union
2	75/F	Distal	A1	9	Present	Bridging	Union
3	59/F	Proximal	A1	**7 (at risk)**	**Present**	**Bridging**	**Union**
4	24/M	Middle	A3	**6 (at risk)**	**Present**	**Non-bridging**	**Nonunion**
5	68/F	Middle	A1	11	Present	Non-bridging	Union
6	20/M	Distal	B2	9	Present	Bridging	Union
7	58/F	Distal	B2	10	Present	Bridging	Union
8	81/M	Middle	A3	12	Present	Non-bridging	Union
9	69/M	Middle	A3	10	Present	Bridging	Union
10	48/F	Proximal	B2	11	Present	Bridging	Union
11	50/F	Middle	A2	11	Present	Non-bridging	Union
12	65/F	Proximal	A1	**6 (at risk)**	**Absent**	**Absent**	**Nonunion**

*F* female; *M* male; *OTA* Orthopaedic Trauma Association; *RUSHU* Radiographic Union Score for HUmeral fractures

### Nonunion prediction

**Fig. 3:**
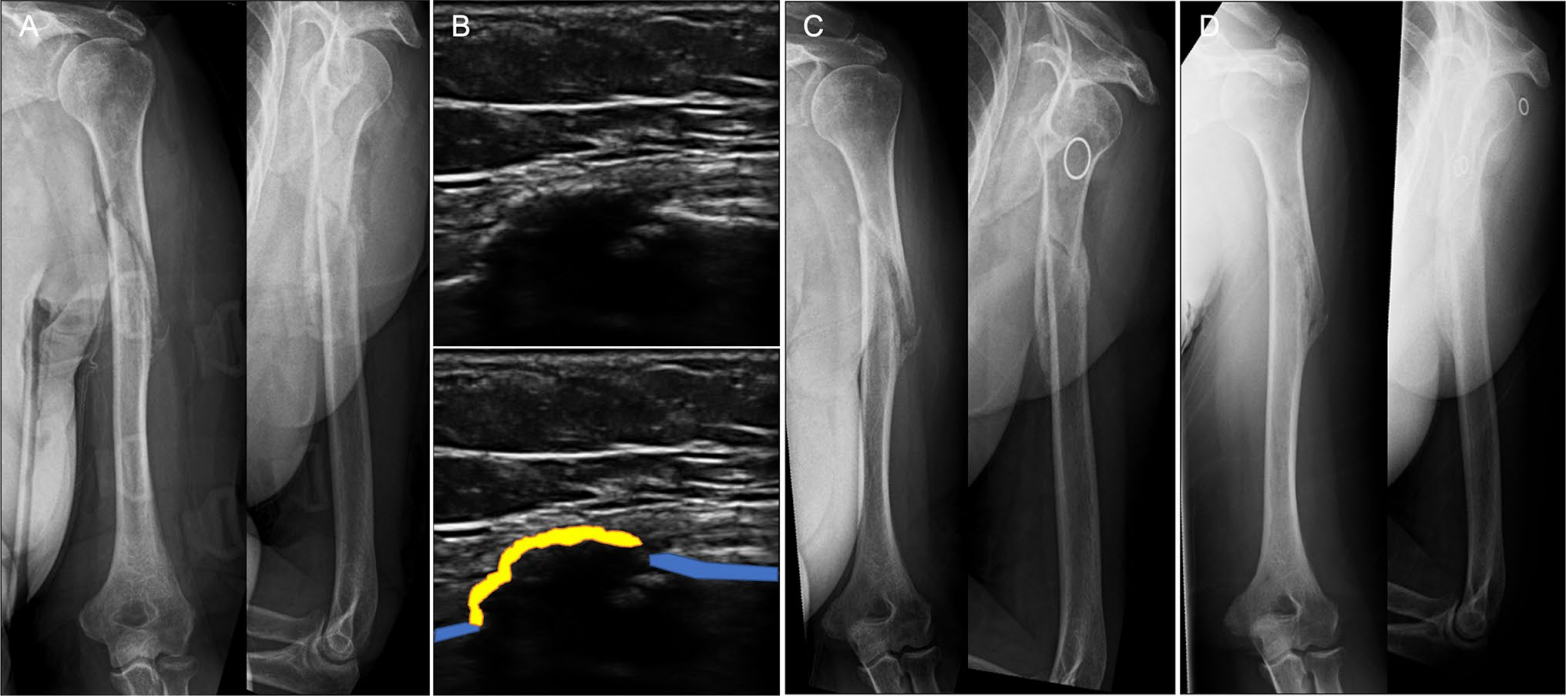
Sequence of images for a 59 year-old female patient with a left humeral shaft fracture: **A** Six-week anteroposterior (AP) and lateral radiographs (Radiographic Union Score for HUmeral fractures = 7); **B** Six-week ultrasound scan image (blue = bone cortex, yellow = sonographic callus); **C** 12-week AP and lateral radiographs (showing union); **D** Eight-month AP and lateral radiographs (showing fracture consolidation)

**Fig. 4:**
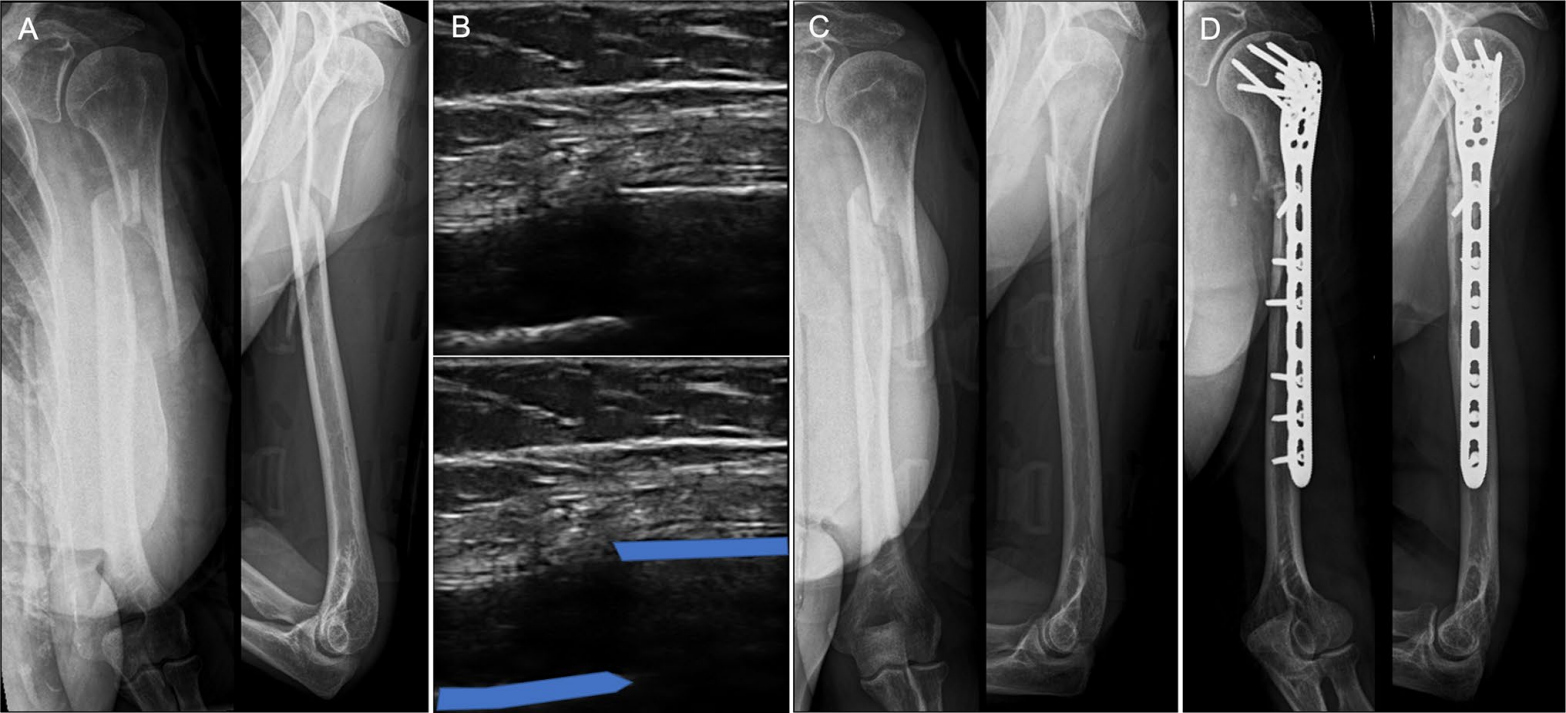
Sequence of images for a 65 year-old female patient with a left humeral shaft fracture: **A** Six-week anteroposterior (AP) and lateral radiographs (Radiographic Union Score for HUmeral fractures = 6); **B** Six-week ultrasound scan image (blue = bone cortex); **C** 12-week AP and lateral radiographs (showing nonunion); **D** AP and lateral radiographs following nonunion surgery (performed at 37-weeks post-injury)

At six weeks post-injury, the absence of SC demonstrated a sensitivity of 50%, specificity 100%, positive predictive value (PPV) 100% and negative predictive value (NPV) 91% in nonunion prediction (overall accuracy 92%; Table 3). The absence of SBC demonstrated a sensitivity of 100%, specificity 70%, PPV 40% and NPV 100% in nonunion prediction (overall accuracy 75%; Table 3). Of three patients identified as being at increased risk of nonunion, based upon reduced radiographic callus formation (RUSHU < 8), one (Patient 3) had SBC on six-week ultrasound and went on to unite (Table 2 and Fig. 3). The other two (Patients 4 and 12) had non-bridging or absent SC, and both developed a nonunion (Table 2 and Fig. 4).

**Table 3:** Accuracy of six-week ultrasound assessment in predicting humeral shaft nonunion

	Union (*n* = 10)	Nonunion (*n* = 2)	
Sonographic callus			
Present	10	1	NPV = 0.91
Absent	0	1	PPV = 1
	Specificity = 1	Sensitivity = 0.5	Accuracy = 0.92
Sonographic bridging callus			
Present	7	0	NPV = 1
Absent	3	2	PPV = 0.4
	Specificity = 0.7	Sensitivity = 1	Accuracy = 0.75

*PPV* positive predictive value; *NPV* negative predictive value

## Discussion

In this prospective pilot study of patients with nonoperatively managed humeral shaft fractures, the assessment of healing using ultrasound imaging was technically feasible, well-tolerated and demonstrated substantial intra- and inter-observer reliability. At the six-week ultrasound assessment, the presence of sonographic callus (SC) in general, and of sonographic bridging callus (SBC) in particular, were found to be accurate predictors of subsequent nonunion. These results suggest that ultrasound is a potentially valuable tool in the early identification of patients at risk of humeral shaft nonunion, particularly those with reduced radiographic callus formation at six weeks post-injury (RUSHU < 8).

Nicholson et al*.* [17] reported the observer agreement for a cohort of 30 patients with non-operatively managed clavicle fractures that underwent sonographic assessment for union. The study documented high levels of reliability for SBC, in terms of intra-observer agreement (*kappa* 0.92) and inter-observer agreement between two (*kappa* 0.82) and four raters (ICC 0.82). However, that study did not include data on the reliability of the presence or absence of SC more generally, with the interpretation of SC considered likely to vary widely and to be of less clinical application. The present study therefore provides useful data confirming the substantial reliability of ultrasound in the context of humeral shaft fractures, while specifically incorporating non-bridging (as well as bridging) SC as part of the rating system.

These data suggest ultrasound assessment at six weeks following a humeral shaft fracture may help predict subsequent nonunion. The relationship between ultrasound and fracture healing has previously been explored following internal fixation of humeral shaft fractures [11], IM nailing of tibial fractures [13–15] and non-operative management of clavicle fractures [17, 18]. Moed et al*.* [14] found that ultrasound performed at six and nine weeks following tibial IM nailing had a 100% sensitivity and 97% PPV to predict subsequent union. Chachan et al*.* [15] determined that ultrasound at fortnightly intervals had a 100% sensitivity and 80% specificity to diagnose union following tibial IM nailing. However, in both studies, it was unclear upon which scan timepoints these data were based [14, 15]. Similarly, although studies investigating the use of Doppler ultrasound noted an association between poor callus vascularity and delayed or nonunion, the timepoints at which these findings had become apparent were unclear and no formal data regarding accuracy were reported [11, 16].

In a cohort of 112 patients with a nonoperatively managed clavicle fracture, Nicholson et al*.* [18] found the absence of SBC had a sensitivity of 94% but a PPV of 41% in predicting subsequent nonunion, comparable to the present study findings (sensitivity 100%, PPV 40%). This illustrates one potential drawback of relying upon SBC alone as an early indicator of nonunion, in that around 30% of patients with nonoperatively managed fractures of the clavicle and humerus appear not to develop SBC at six weeks post-injury, despite eventually going on to unite. The present study also found there was little progression in the proportion of patients with SC or SBC between six and 12 weeks following a humeral shaft fracture. This may suggest that the most substantial production of SC occurs within the first six weeks of fracture, underlining the potential value of ultrasound in the assessment of early fracture healing. In particular, this study found that absence of SBC on six-week ultrasound assessment may be useful in the early identification of nonunion, particularly in those with reduced callus formation on radiographs (RUSHU < 8). Any measures by which patients at risk of nonunion could be identified at an early stage in their non-operative management–and potentially offered surgical fixation–may mitigate the well-documented longer-term negative impact of nonunion [8, 9].

Scanning patients with nonoperatively managed humeral shaft fractures poses various practical issues, yet there are limited details regarding the scanning technique in the existing literature. Maffulli and Thornton [19] performed ultrasound scanning for 24 patients with nonoperatively managed fractures, of which six involved the humeral shaft. Scans were obtained on admission and at three weeks, three months and one year post-injury. The authors acknowledged the heterogeneity of their cohort, and although technical details regarding humeral shaft image acquisition and interpretation were lacking, the study provided useful generic advice regarding ultrasound interpretation. The present study is the first to provide a detailed account of the scanning technique, with suggested acoustic windows for ultrasound assessment. Several practical challenges were encountered over the course of the study. An excessive soft tissue envelope had the propensity to obscure visualisation of the humerus, but it was nonetheless possible to obtain adequate images even in obese patients. There were no morbidly obese patients (BMI > 40 kg/m^2^) in the study cohort, but we would anticipate technical difficulties with the procedure relating to the soft tissue envelope and loss of image resolution associated with increased penetration depth. In some patients, the volume of fracture callus was much larger than the 38 mm ultrasound probe, limiting the ability to obtain a single image frame portraying both fracture cortices with intervening callus (if present). This issue may be more apparent in fractures of the humeral shaft compared to the clavicle or stabilised long-bone fractures, where the higher-strain environment may stimulate increased callus formation [27]. Although scanning through the anterolateral window was universally well-tolerated (and technically more straightforward), scanning via the anteromedial window (which necessitated gentle external rotation and minor shoulder abduction) was sometimes more difficult due to patient discomfort. However, the modest duration of ultrasound scans and overall patient-acceptability make ultrasound a feasible option worthy of further consideration in the outpatient clinic setting.

Su et al*.* [11] performed a prospective study of colour Doppler flow imaging for 65 patients with humeral shaft fractures managed operatively (with an unspecified fixation technique). Scans were obtained weekly between the first and fourth week postoperatively, as well as at the ninth and 15^th^ week. The study focused upon detection of neoangiogenesis and the blood flow resistance index (RI) within the fracture callus, providing insight into the restoration of normal vascularity during postoperative fracture healing. However, Reed et al*.* [28] have demonstrated that nonunions have similar vessel density to normal healing fractures, suggesting that neoangiogenesis per se may not be able to identify nonunions.

Although this represents a prospective proof-of-concept for the ultrasound assessment of humeral shaft fracture healing, we acknowledge the study was not powered to assess the efficacy of ultrasound in nonunion prediction. A much larger cohort (incorporating a larger number of patients with nonunion) would be necessary to determine the accuracy of ultrasound for this purpose. The majority of patients that were approached (73%) declined study participation, which may reflect the fact that the use of ultrasound for this purpose is novel and somewhat experimental. However, this study (the results of which were obviously not available at the time of recruitment) may offer reassurance to patients regarding scan discomfort and duration, thereby potentially improve recruitment rates in future studies. Furthermore, the ability to highlight to patients their potential risk of nonunion at an early stage may help them make informed decisions about their ongoing management. Assessment of microvascular blood flow using Doppler ultrasound may have enhanced our ability to assess callus vascularity, although there is controversy regarding the importance of trends in Doppler parameters (such as the RI and the spectral waveform) in the context of nonunion [11, 16]. It is recognised that the use and interpretation of ultrasound are operator-dependent [29], and while three-dimensional ultrasound reconstruction holds the promise of further improving reliability [30], the requirement for detailed image rendering currently obviates the benefits in terms of immediacy and usability in the outpatient setting.

## Conclusions

Ultrasound assessment of humeral shaft fracture healing was feasible and acceptable from a patient perspective, with substantial intra- and inter-observer reliability. Sonographic callus on six-week ultrasound images demonstrated potential to predict subsequent humeral shaft nonunion. Ultrasound may be useful in the early identification of patients at risk of humeral shaft nonunion, based upon reduced radiographic callus formation at six weeks post-injury (Radiographic Union Score for HUmeral fractures < 8).

## Data Availability

Study data will be made available upon request.
